# Effects of TS-142, a novel dual orexin receptor antagonist, on sleep in patients with insomnia: a randomized, double-blind, placebo-controlled phase 2 study

**DOI:** 10.1007/s00213-022-06089-6

**Published:** 2022-03-17

**Authors:** Makoto Uchiyama, Daiji Kambe, Yumiko Imadera, Yu Kajiyama, Hiroki Ogo, Naohisa Uchimura

**Affiliations:** 1 Department of Psychiatry, Nihon University School of Medicine, 30-1 Oyaguchi-kamicho, Itabashi, Tokyo 173-8610 Japan; 2 Tokyoadachi Hospital, 5-23-20 Hokima, Adachi, Tokyo 121-0064 Japan; 3 Development Headquarters, Taisho Pharmaceutical Co., Ltd, 3-24-1 Takada, Toshima, Tokyo 170-8633 Japan; 4 Department of Neuropsychiatry, Kurume University School of Medicine, 67 Asahi-machi, Kurume, Fukuoka 830-0011 Japan

**Keywords:** Clinical Trial, Insomnia, Orexin, Orexin Receptor Antagonists, ORN0829, Polysomnography, Randomized Controlled Trial, Sleep diary, Sleep Initiation and Maintenance Disorders, TS-142

## Abstract

**Rationale:**

Novel compound with potent antagonistic activity against orexin receptors may be new treatment option for patients with insomnia.

**Objective:**

The aim was to investigate the efficacy and safety of single oral doses of the dual orexin receptor antagonist TS-142 in patients with insomnia.

**Methods:**

This multicenter, double-blind, crossover randomized clinical trial included non-elderly patients with insomnia. Patients were randomized to receive single doses of placebo and TS-142 at doses of 5, 10, and 30 mg in one of four different sequences, with a 7-day washout period between treatments. Primary efficacy endpoints were latency to persistent sleep (LPS) and wake time after sleep onset (WASO) measured by polysomnography.

**Results:**

Twenty-four patients were included (mean age 50.3 ± 10.5 years; mean duration of insomnia 5.71 ± 8.68 years). Least-squares mean differences (95% confidence interval) from placebo in LPS with 5, 10, and 30 mg TS-142 were − 42.38 (− 60.13, − 24.63), − 42.10 (− 60.02, − 24.17), and − 44.68 (− 62.41, − 26.95) minutes, respectively (all *p* < 0.001). Least-squares mean differences (95% confidence interval) from placebo in WASO with 5, 10, and 30 mg TS-142 were − 27.52 (− 46.90, − 8.14), − 35.44 (− 55.02, − 15.87), and − 54.69 (− 74.16, − 35.23) minutes, respectively (all *p* < 0.01). Self-reported aspects of sleep initiation and sleep quality, determined using the Leeds Sleep Evaluation Questionnaire (LSEQ), were also improved with TS-142 administration versus placebo. TS-142 was well tolerated; all adverse events were mild or moderate and none were serious.

**Conclusion:**

Single-dose TS-142 was well tolerated and had clinically relevant effects on objective and subjective sleep parameters in patients with insomnia.

**Clinical Trial registration:**

JapicCTI173570 (www.clinicaltrials.jp); NCT04573725 (www.clinicaltrials.gov).

## Introduction

Insomnia is characterized by nocturnal sleep difficulties despite adequate sleep circumstances which lead to significant daytime impairment over the course of months. Persistent daytime impairments due to chronic insomnia have a negative impact on various aspects of daily life, including school life, social activities, and relationships (Morphy et al. 2007; DiBonaventura et al. 2015; Hägg et al. 2015; Kling et al. 2010; Kyle et al. 2010; Laugsand et al. 2014). Moreover, epidemiological studies have indicated that insomnia is a risk factor for several physical and psychiatric disorders including cardiovascular disease, type 2 diabetes, depression, and others (Khan et al. 2017; Cappuccio et al. 2010; Vgntzas et al. 2009; Baglioni et al. 2011; Freeman et al. 2020). The high prevalence rates reaching 9% to 12% and associated personal and social negative consequences mean that insomnia represents a significant public health problem (Kay-Stacey et al. 2016).

The main goals of insomnia treatment are to ameliorate sleep difficulties which cause poor sleep quantity and quality, and to mitigate insomnia-related daytime impairments (Schutte-Rodin et al. 2008). Treatment options for insomnia include both non-pharmacological and pharmacological approaches; the latter is most widely used, especially in primary care settings. Short-acting benzodiazepine receptor agonists are the most prescribed pharmacological medication used worldwide for the treatment of insomnia (Schutte-Rodin et al. 2008); however, these agents are not strongly recommended in the American Academy of Sleep Medicine (AASM) guideline (Sateia et al. 2017) because they are associated with negative effects, including muscle relaxant and sedative properties leading to an increased trend for falls, injuries, and fractures (Treves et al. 2018), and higher rates of traffic accidents (Agravat 2018). Therefore, there is an unmet need for safer hypnotic agents.

Orexins are neuropeptides that were first discovered in 1998 (de Lecea et al. 1998; Sakurai et al. 1998) and have been shown to play an important role in maintaining wakefulness and regulating the sleep–wake cycle (Mieda 2017; Sakurai 2007; Saper et al. 2005; Kukkonen and Leonard 2014). There are two types of orexin receptors: Orexin-2 receptors (OX_2_) have an important role in sleep regulation, while orexin-1 receptors (OX_1_) act synergistically with OX_2_ to regulate rapid eye movement sleep (Han et al. 2020). Several compounds antagonizing orexin receptors have been developed (Sateia et al. 2017; Janto et al. 2018). Of these, majority including suvorexant, lemborexant, and daridorexant are known to be dual orexin receptor antagonists (DORA); on the contrary, seltorexant is a selective antagonist against OX_2_ (Bonaventure et al., 2015). Suvorexant and daridorexant were reported to antagonize OX_1_ and OX_2_ equally (Cox et al., 2010; Treiber et al., 2017); however, lemborexant was reported to be designed to have a somewhat higher affinity for OX_2_ over the OX_1_ (Beuckmann et al, 2017). In addition to functional antagonist activity in vitro, data for binding potential and dissociation kinetics from orexin receptors, pharmacokinetics profiles, and estimated receptor occupancies are also available; each compounds has its pharmacological and pharmacokinetic characteristics (Gotter et al., 2013; Treiber et al., 2017).

TS-142 is a novel and potent dual orexin receptor antagonist designed to have the pharmacokinetic properties of fast absorption and short plasma half-life. TS-142 has antagonist activities against human OX_1_ and OX_2_ with IC_50_ values of 1.05 nM (OX_1_) and 1.27 nM (OX_2_) (Futamura et al. 2020). In healthy adult subjects, TS-142 reached a maximum concentration within 2.50 h of administration and has an elimination half-life of 1.32—3.25 h when administered orally (Uchiyama et al. 2020). The efficacy such as sleep onset and maintenance and the safety such as next day residual effects of dual orexin receptor antagonist with such pharmacokinetic characteristics are not fully elucidated. This early phase 2 study investigated the efficacy and safety of single oral doses of TS-142 in patients with insomnia.

## Methods

### Study design

**Fig. 1:**
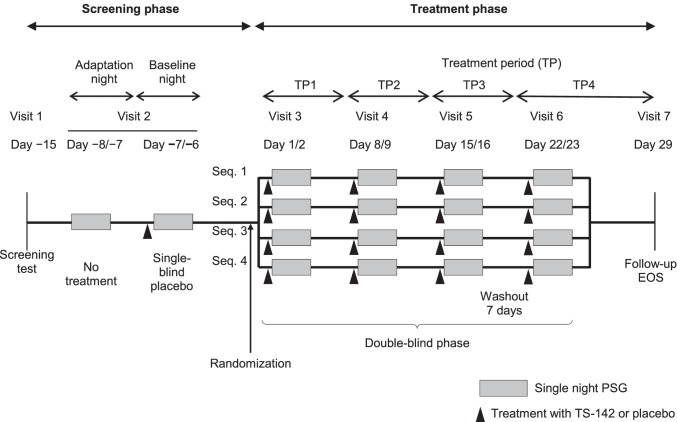
Study design. EOS, end of study; PSG, polysomnography; Seq, sequence

This multicenter (15 medical facilities), randomized, double-blind, placebo-controlled, four-group, four-period crossover, exploratory phase 2 study (JapicCTI173570; NCT04573725) was conducted from July 2017 through February 2019. The study consisted of two consecutive phases: a screening phase including an adaptation night without any treatment and a baseline night with single-blind placebo; and a double-blind treatment phase consisting of four periods in which single doses of TS-142 or placebo were given, followed by a safety follow-up assessment after the final treatment period. All treatments were separated by a 7-day washout period Fig.1). The study protocol was approved by the relevant institutional review boards, and the study was conducted in compliance with the ethical principles outlined in the Declaration of Helsinki and according to Good Clinical Practice. All patients provided written informed consent prior to enrollment in the study.

### Patients

Eligible patients were those aged 20 to 64 years, who had a Diagnostic and Statistical Manual of Mental Disorders, Fifth Edition diagnosis of insomnia based on the investigator-judgment (American Psychiatric Association 2013), who had a total score of ≥ 15 on the Insomnia Severity Index, subjective sleep latency of ≥ 30 min, nocturnal awakening of ≥ 30 min on three or more days per week for 4 weeks prior to the screening phase, daily time in bed of 6.5–9 h, and a bedtime of 21.00–24.00 h at the start of the screening phase. Inclusion criteria for the study were: (1) latency to persistent sleep (LPS) of ≥ 15 min and wake after sleep onset (WASO) of ≥ 45 min on polysomnography (PSG) on the adaptation night; and (2) LPS of ≥ 20 min and WASO of ≥ 60 min on the single-blind placebo-treated night during the screening phase. Patients who met any of the following exclusion criteria were excluded: hypersomnolence disorder, narcolepsy, breathing-related sleep disorders, circadian rhythm sleep–wake disorder, parasomnias, restless legs syndrome, substance/medication-induced sleep disorder, organic brain disease, blood pressure > 160/100 mmHg, history of diabetes mellitus, difficulty sleeping due to concomitant medical conditions, psychiatric diseases, other serious medical conditions, including malignancy, history of serious allergies and/or drug allergy, use of recreational drugs, participation in another clinical trial, previous use of TS-142, women of childbearing potential or who were pregnant or breastfeeding, and men not willing to use appropriate contraceptive methods during the study.

### Randomization

Patients were randomized by study investigators at each participating facility at an intended 1:1:1:1 ratio (block size 4) to four different treatment sequences using a computer-generated list of random numbers provided by an independent company (EPS Systems). Each group received one of the four dosing sequences of TS-142 and placebo using a Williams design: TS-142 5 mg, TS-142 30 mg, TS-142 10 mg, and placebo (Sequence 1); TS-142 10 mg, TS-142 5 mg, placebo, and TS-142 30 mg (Sequence 2); TS-142 30 mg, placebo, TS-142 5 mg, and TS-142 10 mg (Sequence 3); and placebo, TS-142 10 mg, TS-142 30 mg, and TS-142 5 mg (Sequence 4). Treatment sequences were balanced for period and preceding treatment.

### Treatments

Patients received single doses of TS-142 5 mg, TS-142 10 mg, TS-142 30 mg, and matching placebo. Patients slept in the sleep laboratory for 2 consecutive nights (3 days) during the adaptation and baseline nights and for 1 night (2 days) for each treatment period.

Hypnotics were not permitted from 4 weeks prior to the screening phase until the end of the post-study follow up. Dietary supplements with the potential to affect sleep (e.g., those containing melatonin) were prohibited during the screening and treatment phases.

### Endpoints

The primary efficacy endpoints were LPS, which was defined as the time from lights off to the first PSG recording epoch of any continuous sleep that lasted for at least 10 min, and WASO which was measured using overnight PSG. Besides these endpoints, total sleep time (TST), sleep efficiency (SE), and number of awakenings (NAW) were induced as secondary efficacy endpoints using PSG. In addition to PSG outcomes, subjective sleep latency (sSL), subjective TST (sTST), subjective WASO (sWASO), and subjective NAW (sNAW) were also assessed as secondary efficacy endpoints using sleep diaries. The Leeds Sleep Evaluation Questionnaire (LSEQ) is widely used in clinical studies to assess treatment outcomes based on four aspects of subjective sleep quality: ease of getting to sleep (GTS); quality of sleep (QOS); ease of awakening from sleep (AFS); and coordination of behavior following wakefulness (BFW) (Parrott and Hindmarch 1980; Hindmarch 1975). Patients rated their sleep quality on a visual analog scale, where scores less than zero indicated an improvement in sleep quality and scores greater than zero indicated a deterioration in sleep quality. The following safety endpoints were assessed: adverse events, vital signs (body temperature, blood pressure, and pulse rate), 12-lead electrocardiogram (RR, PR, QRS, QT, and normal/abnormal determination), clinical laboratory tests (hematology: white blood cell count, red blood cell count, hemoglobin, hematocrit, platelet count, differential leukocyte count [neutrophils, lymphocytes, monocyte, eosinophils, basophils]; biochemistry: total protein, albumin, albumin/globulin ratio, aspartate aminotransferase, alanine aminotransferase, lactate dehydrogenase, total bilirubin, direct bilirubin, alkaline phosphatase, γ-glutamyl transpeptidase, creatine kinase, blood urea nitrogen, creatine, uric acid, sodium, potassium, chlorine, calcium, phosphorus, total cholesterol, triglyceride, C-reactive protein; urinalysis: specific gravity, pH, protein, occult blood, ketone bodies, bilirubin, urobilinogen, glucose, and sediments), and the Japanese versions of the Karolinska Sleepiness Scale (KSS) (Akerstedt and Gillberg 1990) and the Digit Symbol Substitution Test (DSST) (Weschsler 1939).

Vital sign monitoring, electrocardiogram, and laboratory tests were performed during the study. All adverse events that occurred during the study were recorded by study investigators.

### Assessments

Patients underwent a total of six overnight PSG sessions during the study: at the end of the first week of the screening phase (adaptation night), at the end of the 2-week screening phase (baseline night), and during each of the four treatment periods Fig. 1). The start time for PSG measurement was within ± 30 min of the median bedtime measured for 7 days before the adaptation PSG. Sleep–wake stages in each 30-s epoch on PSG recordings were classified by a blinded central reviewer using version 2.3 of the AASM Manual for the Scoring of Sleep and Associated Events. Parameters determined were LPS, WASO, total recording time, TST, NAW, each sleep stage and SE (TST as a proportion of the total recording time), apnea–hypopnea index, and the periodic limb movement of sleep arousal index. Subjective sleep data were obtained from patient diaries.

Patients completed the LSEQ and Japanese versions of the KSS and DSST during the baseline night and at each of the four treatment periods. A validated Japanese version of the LSEQ was administered just prior to discharge using previously described methods (Hindmarch 1975). The KSS and DSST were completed 9 h after administration of the study drug.

### Statistical analysis

The full analysis set included patients who received at least one dose of study drug and had at least one set of efficacy data. The per-protocol set included patients without any serious protocol violations who had both LPS and WASO data available. The safety analysis set included patients who received at least one dose of study drug and in whom safety endpoints were measured and observed at least once after drug administration.

Patient demographic and clinical data were summarized using descriptive statistics. All efficacy endpoint data are presented as mean ± standard deviation. Least squares mean (LSM) difference compared with placebo and the associated 95% confidence interval (CI) were calculated using a mixed-effects model with treatment group, sequence, and period as fixed effects, and patient as the random effect. Missing data were not imputed because there were no alternative observation data in this single-dosing study. Because the data in this study were not adjusted for multiplicity, all *p*-values were nominal. The two-sided significance level was set to 5%. The detection of dose-dependent effects was conducted using the maximum contrast method with the mean value for each group. Based on the study objective to explore optimal doses of TS-142 for the treatment of insomnia, the sample size was set to 32 with reference to a similar clinical study (De Boer et al. 2018). The mean value and intra-individual error for LPS and WASO were assumed to be identical to those reported in the similar clinical study (De Boer et al. 2018). Under these conditions, the planned sample size would yield approximately 85% power to detect dose-related trends using contrast 5, 1, − 3, and − 3 for placebo, 5, 10, and 30 mg of TS-142 for both primary endpoints. Enrollment was slower than expected; therefore, the study was completed after 24 patients were enrolled, which was considered to be the minimum number necessary to evaluate the effects of TS-142 on LPS and WASO. Statistical analyses were performed using SAS version 9.4 (SAS Institute, Tokyo, Japan).

## Results

### Study population

**Fig. 2:**
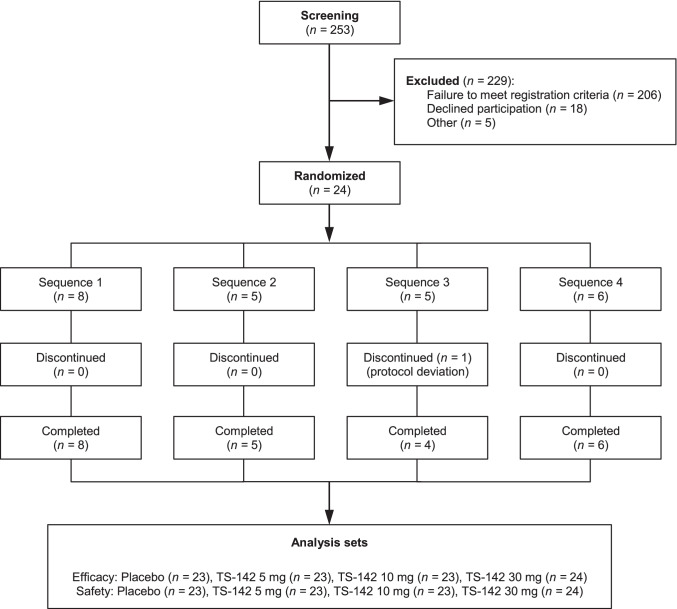
Patient disposition. The orders of administration in each sequence are described in methods.

A total of 253 patients provided written informed consent and were enrolled during the screening phase. Of these, 24 met all of the inclusion criteria and none of the exclusion criteria and were randomized Fig. 2). All randomized patients received at least one dose of the investigational product. One patient was withdrawn from the study due to a protocol deviation after receiving TS-142 30 mg during the first treatment period. The full analysis and safety analysis sets both included 24 patients in the 30 mg TS-142 group and 23 patients in each of the other groups; the per-protocol set included 23 patients. Patient characteristics are shown in Table 1.

**Table 1:** Baseline patient demographic and clinical characteristics (full analysis set)

Variables	Patients (*n* = 24)	
	Mean ± SD	Range
Female, n (%)	10 (41.7)	–
Age, years	50.3 ± 10.5	26–63
Body mass index, (kg/m^2^)	22.28 ± 2.66	16.9–28.5
Duration of insomnia, (years)	5.71 ± 8.68	0.5–43.0
Insomnia severity index [ISI] score	19.9 ± 2.8	16–26
Apnea–hypopnea index [AHI], (/h)	5.24 ± 4.25	0.0–14.8
Periodic leg movement of sleep arousal index, /h	1.97 ± 3.41	0.0–14.7
Polysomnography		
Latency to persistent sleep [LPS], (min)	72.67 ± 59.75	20.0–249.5
Wake time after sleep onset [WASO], (min)	102.88 ± 62.93	60.5–343.0
Sleep diary		
Subjective sleep latency [sSL], (min)	74.8 ± 55.4	5–240
Subjective wake time after sleep onset [sWASO], (min)	104.0 ± 73.5	15–360
Subjective total sleep time [sTST], (min)	301.3 ± 96.9	50–455

Abbreviation: SD, standard deviation

### Efficacy

**Fig. 3:**
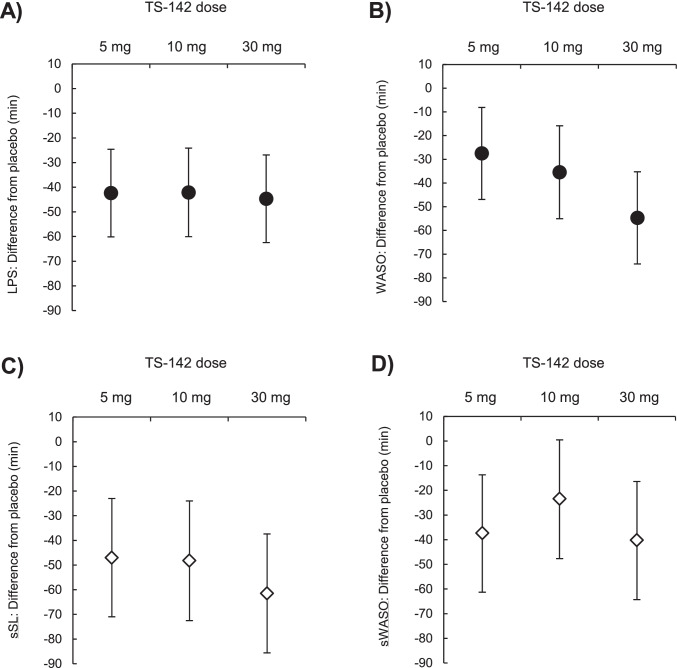
Least-squares mean difference in objective and subjective sleep parameters with TS-142 compared with placebo. Least-squares mean difference in objective (**A** and **B**) and subjective (**C** and **D**) sleep parameters with TS-142 compared with placebo; (**A**) latency to persistent sleep (LPS); (**B**) wake time after sleep onset (WASO); (**C**) subjective sleep latency (sSL); (**D**) subjective wake time after sleep onset (sWASO). Graphs show point estimates (filled circles or opened diamonds) with 95% confidence intervals (vertical bar)

Absolute mean ± standard deviation values for LPS after a single dose of 5, 10, and 30 mg TS-142 and placebo were 12.37 ± 14.56, 10.72 ± 8.72, 7.83 ± 10.32, and 53.57 ± 59.30 min, respectively. Treatment with 5, 10, and 30 mg TS-142 significantly improved LPS. The respective LSM differences (95% CI) from placebo were − 42.38 (− 60.13, − 24.63), − 42.10 (− 60.02, − 24.17), and − 44.68 (− 62.41, − 26.95) minutes (*p* < 0.001 for all doses); a dose-related trend was not observed Table 2,Fig. 3). In contrast to LPS, there was a dose-related trend regarding the effect of treatment on WASO. The LSM differences (95% CI) in WASO between placebo and treatment with 5, 10, and 30 mg TS-142 were − 27.52 (− 46.90, − 8.14), − 35.44 (− 55.02, − 15.87), and − 54.69 (− 74.16, − 35.23) minutes, respectively; the difference between placebo and each dosing group was statistically significant Table 2, Fig. 3). The NAW determined using PSG was not significantly improved compared with placebo at any dose; LSM differences (95% CI) in NAW between placebo and treatment with 5, 10, and 30 mg TS-142 were − 0.6 (− 1.4, 2.5), 0.4 (− 1.6, 2.4), and − 0.3 (− 2.2, 1.7) times, respectively Table 2).

**Table 2:** Changes in objective and subjective sleep parameters with TS-142 compared with placebo (full analysis set)

Sleep parameters	Difference from placebo Point estimate (95% CI)		
	5 mg (***n*** = 23)	10 mg (***n*** = 23)	30 mg (***n*** = 24)
**PSG**			
LPS, (min)	− 42.38 (− 60.13, − 24.63) ***	− 42.10 (− 60.02, − 24.17) ***	− 44.68 (− 62.41, − 26.95) ***
WASO, (min)	− 27.52 (− 46.90, − 8.14) **	− 35.44 (− 55.02, − 15.87) **	− 54.69 (− 74.16, − 35.23) ***
TST, (min)	68.42 (40.20, 96.65) ***	75.44 (46.93, 103.95) ***	97.88 (69.57, 126.19) ***
SE, (%)	14.26 (8.38, 20.14) ***	15.72 (9.78, 21.66) ***	20.39 (14.49, 26.29) ***
NAW	0.6 (− 1.4, 2.5)	0.4 (− 1.6, 2.4)	− 0.3 (− 2.2, 1.7)
Sleep diary			
sSL, (min)	− 47.0 (− 71.0, − 23.0) ***	− 48.2 (− 72.5, − 24.0) ***	− 61.5 (− 85.6, − 37.4) ***
sWASO, (min)	− 37.3 (− 61.0, − 13.7) **	− 23.4 (− 47.3, 0.5)	− 40.2 (− 63.9, − 16.4) **
sTST, (min)	84.3 (50.2, 118.5) ***	71.6 (37.1, 106.1) ***	101.4 (67.0, 135.7) ***
sNAW	− 0.7 (− 1.6, 0.1)	− 0.4 (− 1.2, 0.5)	− 0.4 (− 1.2, 0.5)
LSEQ scores			
GTS	− 25.2 (− 34.5, − 15.9) ***	− 29.2 (− 38.6, − 19.8) ***	− 35.1 (− 44.4, − 25.8) ***
QOS	− 22.9 (− 31.7, − 14.1) ***	− 24.3 (− 33.2, − 15.4) ***	− 24.3 (− 33.1, − 15.5) ***
AFS	− 2.7 (− 11.7, 6.2)	− 1.6 (− 10.6, − 7.5)	6.2 (− 2.7, 15.2)
BFW	− 4.4 (− 12.9, 4.0)	− 3.3 (− 12.0, 5.3)	8.4 (0.0, 16.9)

^*^P < 0.05 versus placebo; **P < 0.01 versus placebo; ***P < 0.001 versus placebo: all *p* values are nominal (not adjusted for multiplicity)

Abbravaations: AFS, awakening from sleep; BFW, behavior following wakefulness; CI, confidence interval; GTS, getting to sleep; LPS, latency to persistent sleep; LSEQ, Leeds Sleep Evaluation Questionnaire; NAW, number of awakenings; PSG, polysomnography; QOS, quality of sleep; s, subjective; SE, sleep efficiency; SL, sleep latency; TST, total sleep time; WASO, wake time after sleep onset

The improvement in sSL and sWASO was significant at all doses of TS-142 compared with placebo with the exception of sWASO after administration of 10 mg TS-142 (LSM differences [95% CI] for sSL for 5, 10, and 30 mg TS-142: − 47.0 [− 71.0, − 23.0], − 48.2 [− 72.5, − 24.0], and − 61.5 [− 85.6, − 37.4] minutes, respectively; LSM differences [95% CI] for sWASO for 5, 10, and 30 mg TS-142: − 37.3 [− 61.0, − 13.7], − 23.4 [− 47.3, − 0.5], and − 40.2 [− 63.9, − 16.4] minutes, respectively) Table 2, Fig.3). There was no significant change in the number of sNAW with TS-142 compared with placebo; LSM differences (95% CI) between placebo treatment with 5, 10, and 30 mg TS-142 were − 0.7 (− 1.6, 0.1), − 0.4 (− 1.2, 0.5), and − 0.4 (− 1.2, 0.5) times, respectively Table 2). The improvement in LSEQ scores for GTS and QOS was significantly with TS-142 treatment compared with placebo. However, there were no significant changes in LSEQ scores for AFS and BFW with TS-142 treatment compared with placebo Table 2).

### Safety

Adverse events by treatment group are summarized in Table 3. No clinically relevant serious treatment-related adverse events were observed, and no patients were withdrawn from the study owing to adverse events. Adverse events tended to be more frequent as the dose of TS-142 increased. The only adverse events that occurred in two or more patients were somnolence and nightmares Table 3). These were considered to have a causal relationship with the study drug. All adverse events were of mild severity, apart from one case of moderate rhabdomyolysis in the TS-142 30 mg group; however, this was considered unrelated to the study drug. No relevant changes in vital signs, electrocardiogram findings, or laboratory tests were documented during the study.

**Table 3:** Adverse events (safety analysis set)

Adverse events	Number of patients (%)			
	Placebo (***n*** = 23)	TS-142		
		5 mg (***n*** = 23)	10 mg (***n*** = 23)	30 mg (***n*** = 24)
Any adverse event	1 (4.3)	2 (8.7)	4 (17.4)	9 (37.5)
Serious adverse event	0	0	0	0
Events				
Somnolence	0	0	1 (4.3)	2 (8.3)
Nightmare	0	0	0	2 (8.3)
Hypnagogic hallucination	0	0	1 (4.3)	1 (4.2)
Muscular weakness	0	1 (4.3)	1 (4.3)	0
Asthenia	0	0	0	1 (4.2)
Feeling abnormal	0	0	0	1 (4.2)
Headache	0	0	1 (4.3)	0
Nausea	0	0	1 (4.3)	0
Nasopharyngitis	1 (4.3)	1 (4.3)	0	0
Arthropod sting	0	0	0	1 (4.2)
Rhabdomyolysis	0	0	0	1 (4.2)

Events are listed using MedDRA/J ver.21.1 preferred terms

Mean ± standard deviation KSS scores did not obviously differ among the placebo (4.6 ± 2.1), 5 mg TS-142 (4.5 ± 2.3), and 10 mg TS-142 groups (5.2 ± 2.0); however, the 30 mg TS-142 group showed slightly higher mean scores (5.9 ± 1.9) Table 4). The mean number and proportion of correct answers on the DSST did not differ between any treatment groups Table 4).

**Table 4:** Summary of next-day residual effects after a single dose of TS-142 based on the KSS and DSST

Evaluation	Placebo (*n* = 23)	TS-142		
		5 mg (***n*** = 23)	10 mg (***n*** = 23)	30 mg (***n*** = 24)
KSS				
Mean score ^a^	4.6 ± 2.1	4.5 ± 2.3	5.2 ± 2.0	5.9 ± 1.9
DSST				
Number of correct answers	52.8 ± 8.6	54.6 ± 7.9	52.0 ± 8.3	49.5 ± 8.5
Proportion of correct answers, %	99.2 ± 1.6	99.6 ± 0.8	99.7 ± 0.7	99.6 ± 0.8

Values are presented as mean ± standard deviation

^a^On a 9-point Likert scale from 1 (very alert) to 9 (very sleepy, fighting sleep), where higher scores indicate more drowsiness

Abbreviations: DSST, Digital Symbol Substitution Test; KSS, Karolinska Sleepiness Scale

## Discussion

TS-142 is a novel dual orexin receptor antagonist characterized pharmacokinetically by fast absorption and short elimination properties (Uchiyama et al. 2020). This is the first clinical study designed to explore a dose range of TS-142 to determine dosing levels that exert sleep-inducing and maintenance effects in patients with insomnia. The effect of TS-142 on sleep was assessed using overnight PSG, sleep diaries, and LSEQ. Subjective and objective next-day residual effects were assessed using the KSS and DSST, respectively. A single dose of TS-142 consistently improved sleep parameters.

This study was conducted by small sample size, however, influences of TS-142 on PSG were relatively clear. One of the significant causes might be the firm inclusion/exclusion criteria for study enrollment. As depicted in Fig. 2, of the 206 patients who were not met either screening criteria, 103 were not fulfill the PSG criteria such as LPS ≥ 15 and WASO ≥ 45 at an adaptation night or LPS ≥ 20 and WASO ≥ 60 min at a baseline night or both. These criteria are generally adopted for PSG studies to exclude effectively patients subjectively complaining insomnia who do not show the insomnia symptom by objectively measured PSG. These PSG criteria might need to assess the effects of hypnotics clearly even though causing a high drop-out rate.

Administration of TS-142 before bedtime induced a sleep promoting effect on patients with insomnia starting at the lowest dose of 5 mg. On the night of placebo administration, the mean LPS was 53.57 min, which represents a typical score for patients who have difficulty in sleep initiation. The LPS was decreased to approximately 8 to 12 min on the night of TS-142 administration. This value range falls within the defined level for a "good sleeper" (Hertenstein et al. 2018), suggesting that a single dose of TS-142 alleviated difficulty with falling asleep. Since type I error potential with multiple testing was not performed, the statistically significant difference reported for LPS must be considered "nominal". However, this effect exceeds the clinically significant threshold (10 min) described in the most recent AASM guideline for the pharmacological management of chronic insomnia Table 2) (Sateia et al. 2017). This improvement in sleep onset may reflect the pharmacokinetic profile of this drug, as a relatively rapid increase in plasma concentration (*t*_max_, range: 0.5 to 2.5 h) was observed in a single ascending dose study of TS-142 (Uchiyama et al. 2020). Although it is difficult to accurately estimate the minimum effective plasma concentration for causing sleep onset through occupancy of both OX_1_ and OX_2_, we speculate that the plasma concentration may have relatively rapidly reached minimal effective concentration after administration. LPS did not demonstrate a clear dose–response trend. It is possible that the time required to reach the minimal effective concentration may be similar in doses ranging from 5 to 30 mg. Both the objective measurement using PSG and the subjective assessment using sleep diaries demonstrated a statistically significant effect at all doses of TS-142 compared with placebo; the effects exceeded the clinically significant time of 10 min in objective measurement, and of 20 min in subjective assessment. In addition, self-rated Visual Analog Scale scores relating to the ease of GTS in the LSEQ showed statistically significant improvement at all doses of TS-142 compared with placebo. These consistent and positive data suggest that TS-142 has sleep-inducing effects and may be a useful treatment option for patients with sleep-onset disorders.

The effect of TS-142 on sleep maintenance (WASO) showed a dose-related trend, which may reflect the pharmacokinetic properties of TS-142. It is assumed that the plasma concentration of TS-142 reaches the minimal effective concentration to exert sleep induction relatively rapidly, and that this concentration is maintained for several hours. We speculate that the period of time that the effective concentration is exceeded increases with increased TS-142 dosing, which would explain the dose-related trend on WASO. Both subjectively and objectively measured WASO demonstrated improved sleep maintenance with TS-142 treatment compared with placebo. Therefore, TS-142 is expected to be an effective treatment option in patients experiencing certain sleep maintenance disturbances.

There was no apparent change in NAW after treatment with TS-142 at any dose whether it was objectively (PSG) or subjectively (sleep diary) evaluated. These data suggest that TS-142 does not have a sufficient inhibitory effect on the endogenous transition from non-rapid eye movement or rapid eye movement sleep to wakefulness as observed in clinical studies of other dual orexin receptor antagonists (Herring et al. 2012, 2016). However, one must carefully consider NAW in the context of other symptoms, especially when subjectively evaluated. Although NAW itself may negatively impact sleep quality and daytime function in patients with insomnia, an epidemiological study indicated that individuals with NAW who did not have any other symptoms of insomnia, including difficulty in resuming sleep, rarely reported daytime impairment (Ohayon et al. 2010). Therefore, the treatment effect on NAW should be considered along with the effects on other sleep properties, such as difficulty resuming sleep and subjective sleep quality, rather than on NAW alone.

The next-day residual effects of hypnotics are a concern, as they may have a negative impact on daily life, such as impaired driving or increased risk of falling. Next-day residual effects are often assessed by KSS, DSST, and the occurrence of somnolence as reported by investigators and participants. In this study, no pronounced score changes were observed for KSS or DSST compared with placebo, especially at lower doses; the number of patients who reported somnolence was low. These data indicate that TS-142 may have a low incidence of next-day residual effects when used within the therapeutic dose range. In addition, these data may support the idea that next day residual effects can be avoided by treating with orexin receptor antagonists, such as TS-142, that have a short half-life. However, further clinical studies with a larger patient population are necessary to further elucidate the extent of next-day residual effects with TS-142 treatment.

The frequency of adverse events in this study was relatively low and the tolerability of TS-142 was favorable. Although we observed an increased frequency of adverse events with increased dose, data from larger patient groups are needed to better understand this relationship. One patient had two episodes of hypnagogic hallucinations of mild severity. Such adverse events are consistent with the known pharmacology of dual orexin receptor antagonists, having been reported in patients treated with other drugs in this class (Herring et al. 2012; Murphy et al. 2017). There were no adverse events of sleep paralysis, excessive daytime sleepiness, or cataplexy-like symptoms. Further studies are needed to accurately determine the dose of TS-142 at which the incidence of mechanism of action-associated adverse events are minimized.

This study had several limitations. First, elderly patients and those with heterogeneous disease backgrounds were excluded; therefore, future studies are needed to assess the effects of TS-142 on these populations. Second, this was a single-dose study, thus the sustained efficacy in repeated dosing of TS-142 needs to be evaluated. Finally, a relatively small number of patients completed this study due to the relatively high screen fail rate (greater than 90%). Therefore, the generalizability of this study, as well as the safety findings, is limited. Therefore, additional larger studies will be needed to fully evaluate the effects of both single and repeated doses of TS-142 on sleep effects.

In conclusion, a single oral administration of TS-142 to insomnia patients showed clinically significant improvement in both objective and subjective evaluations of sleep. A single dose of TS-142 was well tolerated.

## Data Availability

Aggregated data supporting the findings of this study are presented in this manuscript. Currently, there are no plans to share individual participant data publicly, so supporting data are not available.
